# The emerging landscape of brain glycosylation: from molecular complexity to therapeutic potential

**DOI:** 10.1038/s12276-025-01560-8

**Published:** 2025-10-14

**Authors:** Youngsuk Seo, Ji Eun Park, Jae Young Yu, Boyoung Lee, Jong Hyuk Yoon, Hyun Joo An

**Affiliations:** 1 Center for Memory and Glioscience, Life Science Institute, Institute for Basic Science, Daejeon, Republic of Korea; 2 Asia-Pacific Glycomics Reference Site, Daejeon, Republic of Korea; 3 Graduate School of Analytical Science and Technology, Chungnam National University, Daejeon, Republic of Korea; 4 Neurodegenerative Diseases Research Group, Korea Brain Research Institute, Daegu, Republic of Korea

**Keywords:** Glycobiology, Glycosylation

## Abstract

Glycosylation functions as a pivotal posttranslational modification in proteins and as a distinct biosynthetic process in lipids. In the brain, it plays essential roles in development, function and homeostasis by modulating protein folding, receptor trafficking and intercellular communication. Although glycans constitute less than 1% of the brain’s mass, their impact is disproportionately profound. Recent technological advances have uncovered the essential contributions of both protein- and lipid-bound glycans, including N-glycans, O-glycans and gangliosides, to brain physiology and disease. Here we explore the emerging landscape of brain glycosylation, highlighting its distinct roles in neurodevelopment, synaptic organization and immune regulation. Aberrant glycosylation has been implicated in neurodegenerative diseases (for example, Alzheimer’s and Parkinson’s), psychiatric disorders (for example, depression and schizophrenia) and neurodevelopmental conditions (for example, autism spectrum disorders, attention deficit hyperactivity disorder and dystroglycanopathies). We summarize recent breakthroughs in glycomics technologies, including glycan enrichment, liquid chromatography–tandem mass spectrometry, MALDI-based imaging mass spectrometry and high-throughput omics, which enable molecular and spatial mapping of brain glycosylation. Artificial-intelligence-driven bioinformatics and multi-omics integration are rapidly opening new avenues for deciphering glycan-mediated regulation in brain health and disease. Together, these developments position brain glycosylation as a transformative frontier in neuroscience, with the potential to yield novel diagnostic biomarkers and therapeutic strategies for complex brain disorders.

## Brain glycosylation shapes development and disease mechanisms

The human brain is a complex organ with over 100 billion cells, including neurons and glial cells. It controls thoughts, emotions and actions through intricate communication systems. This Review explores glycosylation, a process where sugars attach to proteins and lipids, which is crucial for brain function but not well understood. Researchers have used advanced techniques such as mass spectrometry to study glycosylation in the brain. Studies have shown that glycosylation influences brain development, neuronal communication and disease mechanisms. For example, changes in glycosylation have been associated with Alzheimer’s and Parkinson’s diseases. The study highlights the importance of understanding glycosylation for developing new treatments. The researchers conclude that glycosylation is a key player in brain health and disease. Future research could lead to new diagnostic tools and therapies for brain disorders by focusing on glycosylation patterns.

This summary was initially drafted using artificial intelligence, then revised and fact-checked by the author.

## Introduction

The human brain is an extraordinarily complex organ composed of more than 100 billion specialized cells, including neurons and glial cells^1^. It orchestrates cognition, emotion and behavior through intricate molecular signaling networks that govern both intracellular and intercellular communications^2,3^. Disruption of these systems, such as neurotransmitter pathways (for example, glutamatergic, γ-aminobutyric acid (GABA)ergic and dopaminergic), neurotrophic factor signaling and synaptic plasticity mechanisms, underlies a wide spectrum of brain diseases, including neurodegenerative, neurodevelopmental and psychiatric disorders^4–6^.

Among the diverse molecular regulators, posttranslational modifications play essential roles in modulating protein function, subcellular localization and intracellular signaling pathways, as well as in intercellular communication^7–11^. In particular, glycosylation stands out as a critical and dynamic posttranslational modification in the brain^12–16^. It regulates receptor trafficking, synaptic plasticity and neuron–glia interactions^17^. Despite its biological importance, brain glycosylation has remained one of the least explored molecular features of the central nervous system, largely due to its structural heterogeneity, low abundance in neural tissues and the historical lack of suitable analytical tools and reliable databases.

Over the past decade, molecular neuroscience has made substantial progress. Early studies focused narrowly on individual molecules or pathways, but recent advances in high-throughput omics technologies such as genomics, transcriptomics, proteomics and metabolomics have broadened our understanding of the molecular landscape of the brain^18–20^. Within this expanding framework, glycosylation is emerging as a crucial yet underexplored layer of regulation, with implications for both normal brain function and disease^17^.

Recent advances in mass spectrometry (MS) have begun to overcome prior limitations, enabling high-sensitivity, high-resolution profiling of glycosylation in brain tissues^12,21–24^. Improved sample preparation protocols now allow efficient extraction and enrichment of glycans, overcoming challenges posed by the biochemical complexity of the brain^25,26^. In particular, the integration of liquid chromatography with MS (LC–MS) or tandem MS (LC–MS/MS) has become a powerful platform for structural characterization, allowing detailed analyses of glycan types, monosaccharide composition, linkage patterns and isomeric variants^12,13,27,28^. These platforms also facilitate the analysis of glycan-conjugated molecules, including glycoproteins and glycolipids, two structurally and functionally distinct types of brain glycoconjugate^13,26^. This integrative approach enables the identification of both specific carrier molecules and their associated glycosylation patterns, offering insights into their roles in brain development, synaptic organization and disease mechanism.

Glycomics, glycoproteomics and glycolipidomics, when integrated with other omics platforms such as genomics, transcriptomics and proteomics, offer powerful tools for dissecting the molecular underpinnings of brain function and dysfunction^22,29^. Emerging evidence links altered glycosylation to key disease mechanisms such as neuroinflammation, impaired synaptic signaling and abnormal cell–cell interactions^17,30–32^. These findings underscore the relevance of brain glycosylation not only as a fundamental biological process but also as a promising source of diagnostic biomarkers and therapeutic targets.

In this Review, we highlight recent advances in brain glycosylation research with a particular focus on the distinct functional roles of protein-bound and lipid-bound glycans, their dysregulation in various neurological and psychiatric conditions, and the analytical breakthroughs that have enabled these discoveries possible. We further explore how these developments set the stage for glycosylation-based therapeutic strategies in brain disorders, especially in the era of integrative omics and artificial intelligence (AI)-driven biomedical research.

## Glycosylation overview and its role in brain function

Glycosylation is a fundamental biosynthetic modification catalyzed by hundreds of glycosidases and glycosyltransferases, generating a vast array of protein-bound and lipid-bound glycoforms including N-glycans, O-glycans and glycolipids^9,13,33^. In the brain, glycosylation contributes to homeostasis by regulating protein folding, stability and intracellular communication^34^. It is particularly vital during neurodevelopment, where glycan modifications of neural cell adhesion molecules guide neuronal differentiation, migration and synaptic plasticity^35,36^. Through these mechanisms, brain glycosylation supports neural circuit formation and connectivity, influencing interregional signaling and brain-controlled physiological functions^35,37^. Collectively, glycosylation acts as a crucial molecular regulator linking neurodevelopment and neural network organization. In the following subsections, we introduce the major types of glycosylation in proteins and lipids, highlighting their specific functions in brain homeostasis as elucidated by recent studies.

### Protein-bound glycosylation

**Fig. 1:**
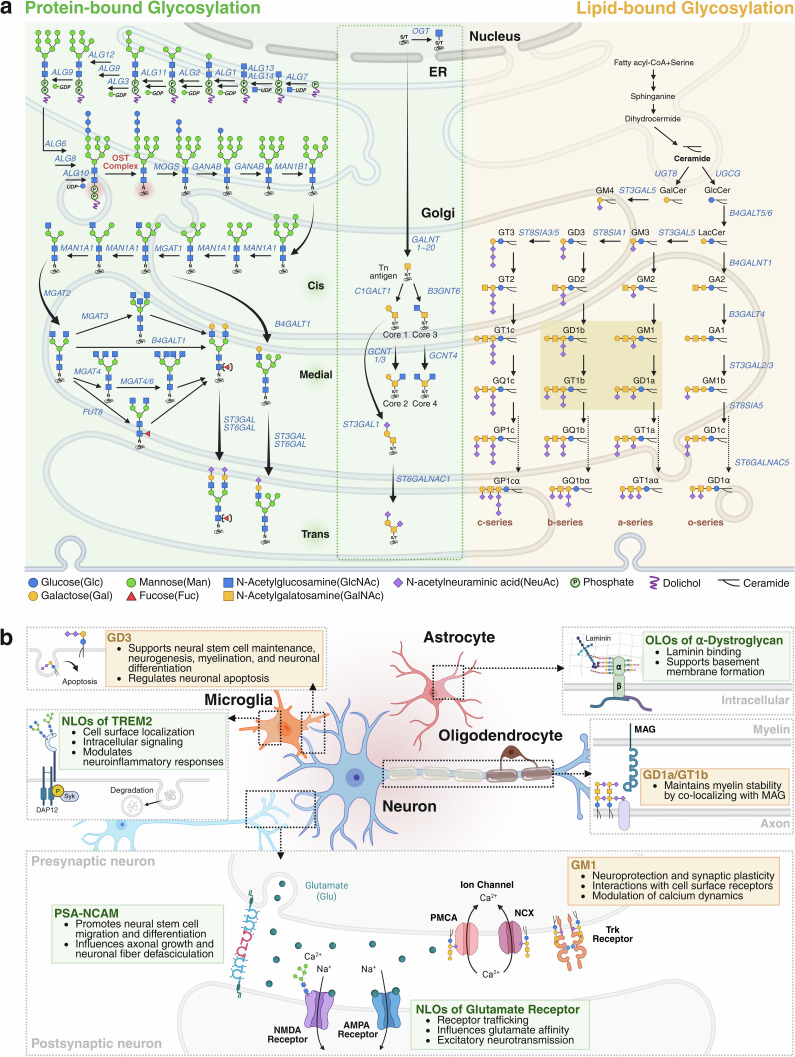
Overview of glycosylation pathways and their functions in the brain. **a** Glyco-moieties on both proteins and lipids are generated through the enzymatic actions of various glycosyltransferases and glycosidases, with the corresponding enzyme genes indicated for each pathway. As illustrated in the green box, protein-bound glycosylation—including N-glycosylation, mucin-type O-GalNAcylation (extending from the nucleus to the Golgi, indicated by a dashed line) and O-GlcNAcylation (in the nucleus)—are synthesized through stepwise enzymatic modifications. Concurrently, gangliosides—the major form of lipid-bound glycosylation—are synthesized from ceramide and further diversified into distinct series by glycosyltransferases, primarily sialyltransferases (depicted in the light-yellow box). The most abundant brain gangliosides (GM1, GD1a, GD1b and GT1b) are highlighted in the dark-yellow box. **b** Glycosylation regulates the function of brain cells and molecules essential for neural communication and homeostasis. Neuronal glycoproteins such as polysialylated (PSA)-NCAM and glutamate receptors modulate synaptic development and plasticity. TREM2 glycosylation enhances microglial signaling, while O-glycosylation of α-DG maintains interactions among neurons, glia and the ECM. N-linked and O-linked oligosaccharides (NLOs and OLOs) indicate N- and O-glycans. In parallel, gangliosides contribute to synaptic plasticity (GM1), myelin stability (GD1a and GT1b) and neurogenesis (GD3). PCMA, plasma membrane Ca^2+^ ATPase; NCX, Na^+^/Ca^2+^ exchanger. Created in BioRender. Park, J. (2025) https://BioRender.com/ol8zb5e.

Protein-bound glycosylation is broadly classified into N-glycosylation and O-glycosylation^9^. The latter encompasses both mucin-type O-glycosylation (O-GalNAcylation) and O-GlcNAcylation^9^. Generally, N-glycosylation is initiated in the endoplasmic reticulum (ER) (Fig. 1a), where a precursor oligosaccharide is assembled on a dolichol phosphate lipid carrier using nucleotide sugar donors such as uridine diphosphate (UDP)-*N*-acetylglucosamine (UDP-GlcNAc). This oligosaccharide is then transferred en bloc to specific asparagine (Asn) residues within the Asn-X-Serine/Threonine (Ser/Thr) motif of nascent proteins, where ‘X’ can be any amino acid except proline (Pro). Subsequent processing in the Golgi apparatus involves the stepwise addition and trimming of monosaccharides, generating structurally diverse N-glycans.

By contrast, O-GlcNAcylation occurs in the nucleus, where a single GlcNAc residue is attached to serine or threonine residues by O-GlcNAc transferase (OGT)^9^ (Fig. 1a). Unlike the complex and branched nature of other O-glycans, O-GlcNAcylation is often dynamic and reversible, analogous to phosphorylation in cellular signaling^38^. Mucin-type O-glycosylation (O-GalNAcylation) is initiated in the Golgi apparatus by a family of 20 polypeptide N-acetylgalactosaminyltransferases (GALNTs), which transfer a GalNAc residue to serine or threonine residues^39^ (Fig. 1a). This initial GalNAc can be further elongated by various glycosyltransferases, generating structurally diverse and often tissue-specific O-glycan chains^26^. In addition to these major forms, several other O-glycan modifications such as O-mannosylation and O-fucosylation are also relevant in brain glycosylation^40,41^. These modifications are catalyzed by enzymes in the Golgi that use guanosine diphosphate (GDP)-linked sugar donors (for example, GDP-mannose and GDP-fucose). They are frequently found in brain-associated proteins, including extracellular matrix (ECM) components, α-dystroglycan (α-DG) and thrombospondins, and have been implicated in neuronal development and signaling^40–42^.

Recent research reveals that protein-bound glycosylation fine-tunes neurocellular communication by regulating neuronal organization, synaptic signaling and glial-mediated immune responses^43–46^ (Fig. 1b). A well-studied example of protein-bound glycosylation is polysialylated neural cell adhesion molecule (PSA-NCAM), which promotes neural stem cell migration and differentiation^43,44^ (Fig. 1b). During the development of the central nervous system, PSA-NCAM is highly expressed, influencing axonal growth, neuronal fiber defasciculation and cell migration^44^. In adulthood, its expression is primarily restricted to the mouse olfactory bulb, rat hippocampus and hypothalamus to retain the ability to generate neurons or to exhibit high neuronal activity^44^. Another example is the glutamate receptor subunit GluN1, where the removal of N-glycans alters glutamate sensitivity, implicating N-glycosylation in receptor trafficking and excitatory neurotransmission^45^ (Fig. 1b). In addition, N-glycosylation of triggering receptor expressed on myeloid cells 2 (TREM2), a microglial transmembrane glycoprotein, is essential for cell surface localization and intracellular signaling, thereby contributing to neuroimmune homeostasis^46^46 (Fig. 1b). In the context of O-glycosylation, α-DG, a membrane glycoprotein, carries O-mannosyl glycans that facilitate ECM binding^47^. During development, these glycan–ECM interactions guide neuronal migration and axon pathfinding, with laminin binding to O-mannosylated α-DG playing a key role in basement membrane formation^47,48^ (Fig. 1b). The glycosylation of glutamate receptors, including α-amino-3-hydroxy-5-methyl-4-isoxazolepropionic acid (AMPA) and *N*-methyl-D-aspartate (NMDA) receptors, as well as NCAM, TREM2 and α-DG, emphasizes the central role of protein-bound glycans in maintaining neural function, modulating inflammation and orchestrating developmental processes.

### Lipid-bound glycosylation

Lipid-bound glycosylation forms glycolipids such as gangliosides, globosides and cerebrosides, which exert their functions at the membrane interface by stabilizing receptor complexes, modulating ion flux and signaling cascades, and facilitating axon–glia interactions essential for long-term neuronal integrity^13,49^. Gangliosides, a major class of glycosphingolipid composed of ceramide and sialylated glycan moieties, are the most abundant brain glycolipids^13,49^. Ganglioside synthesis begins with ceramide formation in the ER, followed by sequential sugar addition in the Golgi via specific glycosyltransferases (Fig. 1a), resulting in structurally diverse species, including isomeric forms^13,49^.

During brain development, ganglioside pattern and expression undergoes a marked transition from simple embryonic forms such as GM3 and GD3 to complex adult forms such as GM1a, GD1a, GD1b and GT1b, for which GM3 and GD3 serve as biosynthetic precursors^49,50^. In the adult brain, four major gangliosides—GM1, GD1a, GD1b and GT1b—make up over 90% of the total brain gangliosides^49^ (Fig. 1a). Among these, GM1 is a key ganglioside with well-established roles in neuroprotection and synaptic plasticity^49^. It is highly enriched in neuronal membranes, where it interacts with a variety of cell surface receptors, especially those involved in neurotrophic and synaptic signaling, including neurotrophic factors, neurotransmitter receptors and ion channels^49,51,52^. In particular, GM1 enhances neuroprotective signaling by stabilizing the interaction between Trk receptors and nerve growth factor, thereby promoting neuronal survival and synaptic plasticity^49,51^ (Fig. 1b). In addition, GM1 modulates calcium dynamics through its interaction with ion channels^49^. In a β-galactosidase-deficient mouse model, Alessandra d’Azzo’s group demonstrated that GM1 accumulation at ER–plasma membrane junctions enhances its interaction with phosphorylated NMDA^52^. This interaction leads to increased Ca²⁺ influx and extracellular signal-regulated kinase (ERK) activation, ultimately resulting in excessive dendritic spine formation without enhancing synaptic connectivity.

Beyond GM1, other major gangliosides such as GD1a and GT1b play essential roles in maintaining myelin stability, which is critical for efficient signal transmission and long-term neuronal integrity^49,53^. In the adult mouse brain, these gangliosides are highly enriched in myelinated tracts including the corpus callosum and corticospinal tract, where they co-localize with myelin-associated glycoprotein (MAG)^53^ (Fig. 1b). This spatial association suggests their function as axonal receptors that contribute to myelin sheath stabilization. During brain development, GD3 ganglioside is particularly important as it supports neural stem cell maintenance, neurogenesis, myelination and neuronal differentiation^54,55^ (Fig. 1b). It also regulates neuronal apoptosis during developmental pruning, ensuring appropriate cell turnover and survival^55^. Collectively, lipid-bound glycosylation is fundamental to brain function. Through its regulation of membrane organization, cell signaling and neural connectivity, it orchestrates key processes such as neuronal development, synaptic plasticity and neuroprotection.

## Clinical implications of brain glycosylation

To better understand the etiology and pathology of neurological and psychiatric disorders, recent studies have increasingly focused on glycosylation patterns in the brain and their regulatory mechanisms^31,56,57^. Accumulating evidence highlights the involvement of glycosylation in major neurodegenerative diseases such as Alzheimer’s disease (AD) and Parkinson’s disease (PD), where aberrant glycosylation contributes to protein misfolding (for example, tau or α-synuclein aggregation) and impaired synaptic plasticity^31,58–63^. Moreover, alterations in brain glycosylation have also been linked to psychiatric disorders such as schizophrenia, major depressive disorder (MDD) and post-traumatic stress disorder (PTSD)^57,64–66^. These findings suggest that glycosylation may play broader roles in modulating synaptic integrity, neural communication and immune balance across a range of brain disorders. In this section, we review recent research highlighting the clinical relevance of brain glycosylation. Special emphasis is placed on both protein- and lipid-bound glycosylation, as they relate to neurological, psychiatric and neurodevelopmental diseases. A summary of disease-associated glycosylation changes is provided in Table 1 (protein-bound) and Table 2 (lipid-bound).

**Table 1:** Brain disorders and associated changes in protein-bound glycosylation.

Brain disorders	Species; sample type	Analytical tools	Glycosylation types	Description	Reference (number, year)
Alzheimer’s disease (AD)	Human; brain tissue	Histochemical staining	α2,6-Sialylation ↑	In patients with AD, microglial N-glycosylation is marked by increased α2,6-sialylation, particularly near Aβ plaques.	^70^, 2024
		Glycoproteomics (LC–MS)	N-glycan sialylation ↓	Protein sialylation is reduced in AD brains, with a marked decrease in highly branched and elongated sialylated N-glycans.	^71^, 2024
		Immunofluorescence	Polysialylation ↓	In human AD brains, PSA-NCAM expression is substantially reduced in layers II and V of the entorhinal cortex.	^62^, 2018
		Western blot	Polysialylation ↑	PSA-NCAM expression is elevated in the AD hippocampus, particularly in the dentate gyrus, and correlates with disease severity.	^72^, 2004
		Glycoproteomics (LC–MS)	Sialofucosylation ↓ High-mannose ↑	AD brains show reduced sialofucosylated N-glycans in cortical and increased high-mannose glycans in noncortical regions.	^56^, 2022
	Human; CSF	Glycomics (MALDI–MS)	Sialylation ↓ High-mannose ↑ Bisecting ↑ Core-fucosylated ↑	In patients with AD, CSF glycans show increased high-mannose and bisecting core-fucosylated N-glycans, along with reduced sialylation of both N- and O-glycans.	^74^, 2024
		Glycomics (LC–MS)	Bisecting GlcNAc ↑ Lewis X ↑	AD CSF shows altered N-glycosylation with increased bisecting GlcNAc and Lewis X.	^58^, 2020
	Human; blood	Glycomics (LC–MS)	N-glycosylation ↓	Blood N-glycan profiling in AD reveals globally reduced N-glycosylation.	^68^, 2025
Parkinson’s disease (PD)	Human; brain tissue	Glycomics (LC–MS)	O-glycan sialylation ↑ O-glycan sulfation ↓	In PD striatum, O-glycans show increased sialylation and reduced sulfation.	^73^, 2021
	Human; serum	Glycomics (MALDI–MS), glycoproteomics (LC–MS)	Core fucosylation ↑ Sialylation ↑ Bisecting GlcNAc ↑	In PD serum, 36 specific glycoproteins exhibit increased N-glycosylation with core fucosylation, sialylation and bisecting GlcNAc.	^61^, 2022
	Human; urine	Glycomics and glycoproteomics (LC–MS)	Biantennary galactosylation ↓	In PD urine, biantennary galactosylated N-glycans are substantially decreased.	^75^, 2023
Depression	Human; EV	Lectin blotting	Sialylated vWF ↓	Plasma EVs from patients with MDD show reduced WGA-binding sialylated vWF, suggesting a loss of terminal GlcNAc and sialic acid.	^92^, 2024
	Human; plasma	Lectin microarray	Sialylation (Sia-α2-6Gal/GalNAc) ↓	In MDD patient plasma, glycosylation is altered with reduced Sia-α2-6Gal/GalNAc structures.	^95^, 2018
	Mouse; brain tissue	Glycoproteomics (LC–MS)	Bisecting ↓	Acute and chronic stress-induced depression models show altered hippocampal N-glycosylation, notably reduced bisecting glycans.	^57^, 2025
		Glycoproteomics (LC–MS)	O-GlcNAcylation ↑	Increased O-GlcNAcylation in astrocytes of the mPFC enhances susceptibility to depressive-like behaviors.	^65^, 2023
PTSD	Rat; brain tissue	Glycomics (Ultra Performance Liquid Chromatography (UPLC)-Fluorescence (FLR) detector)	High branching ↓ Sialylation ↓	Trauma reduces high-branching and sialylated N-glycans in the PFC of stress-susceptible rats.	^64^, 2020
Schizophrenia (SCZ)	Human; serum	Glycomics (High-Performance Liquid Chromatography (HPLC)-FLR)	Polylactosaminylation ↑ SLex-containing ↑	In patients with SCZ, serum shows increased polylactosaminylated and SLex-containing N-glycans, whereas CSF N-glycans show reduced bisecting and sialylation.	^96^, 2010
	Human; CSF		Bisecting ↓Sialylation ↓		
ADHD	Human; serum	Lectin microarray, glycomics (MALDI–MS)	Antennary fucosylation↑ Bisecting ↓ α2-3 Sialylation ↓	ADHD patient serum shows increased antennary fucosylation with decreased bisecting GlcNAc and α2-3 sialylated N-glycans.	^98^, 2023

**Table 2:** Brain disorders and associated changes in lipid-bound glycosylation.

Brain disorders	Species; sample type	Analytical tools	Glycosylation types	Description	Reference (number, year)
Alzheimer’s disease (AD)	Human; brain tissue	Lipidomics (MALDI–MS)	GM1, GD1, GT1	Ganglioside nanoclusters (Aβ–ganglioside complexes) in the precuneus promote Aβ fibril formation and contribute to AD pathology.	^76^, 2024
		Tissue imaging (MALDI–MSI)	GM1 d20:1/18:1 ratio ↓	GM1 d20:1/18:1 ratio is decreased in the dentate gyrus and entorhinal cortex of AD brains, while GM1 and GM3 are enriched around Aβ plaques.	^78^, 2024
		Glycolipidomics (LC–MS)	GM2, GM3, GQ3 ↑ GD1a, GT1b ↓	AD patients show elevated simple gangliosides (GM2, GM3 and GQ3) and reduced complex gangliosides (GD1a and GT1b) in the inferior frontal gyrus.	^80^, 2023
	Mouse brain; brain tissue	Tissue imaging (MALDI–MSI)	GM2, GM3↑	Increased GM2 and GM3 localized within β-amyloid plaques in cortex and dentate gyrus of APP/PS1 mice.	^79^, 2025
		Western blot	GQ1b ↓	The hippocampus of 3xTg-AD mice shows a substantial reduction in GQ1b levels, along with lower brain-derived neurotrophic factor (BDNF) and higher Aβ and phosphorylated tau	^59^, 2019
		Immunofluorescence, western blot	GM1 ↑	Elevated GM1 in AD mouse brain promotes Aβ generation and cognitive decline.	^60^, 2023
Parkinson’s disease (PD)	Human; brain tissue	Glycolipidomics (LC–MS)	GM1, GM2, GM3, GD2, GD3 ↑	GM1, GM2, GM3, GD2 and GD3 are broadly elevated in PD-GBA brains, with the strongest increases observed in the MTG, CG and striatum.	^81^, 2022
		Lipidomics (LC–MS)	Lysosomal GlcCer ↑	Loss-of-function mutations in ATP10B disrupt GlcCer and phosphatidylcholine (PC) export from lysosomes, driving ganglioside dysregulation in PD.	^82^, 2020
	Mouse brain; brain tissue	Immunohistochemistry high-performance thin-layer chromatography (HPTLC)	GM1 ↓	GM1 is reduced in the PD brain, especially in the SNpc and occipital cortex, contributing to both motor and nonmotor symptoms.	^63^, 2020
Epilepsy	Human; brain tissue	Glycolipidomics (LC–MS)	GD1b, GQ2, GT1–3 ↑ Modified gangliosides	Elevated GQ1b, GQ2, GT1–3 and fucosylated/O-acetylated gangliosides characterize the ganglioside profile of temporal lobe epilepsy.	^83^, 2022
Depression	Mouse; brain tissue	Immunohistochemistry	GD3 ↓	GD3 deficiency impairs neurogenesis in the subventricular zone (SVZ) and dentate gyrus (DG), contributing to depression-related behavioral deficits.	^89^, 2024

### Neurological disorders

**Fig. 2:**
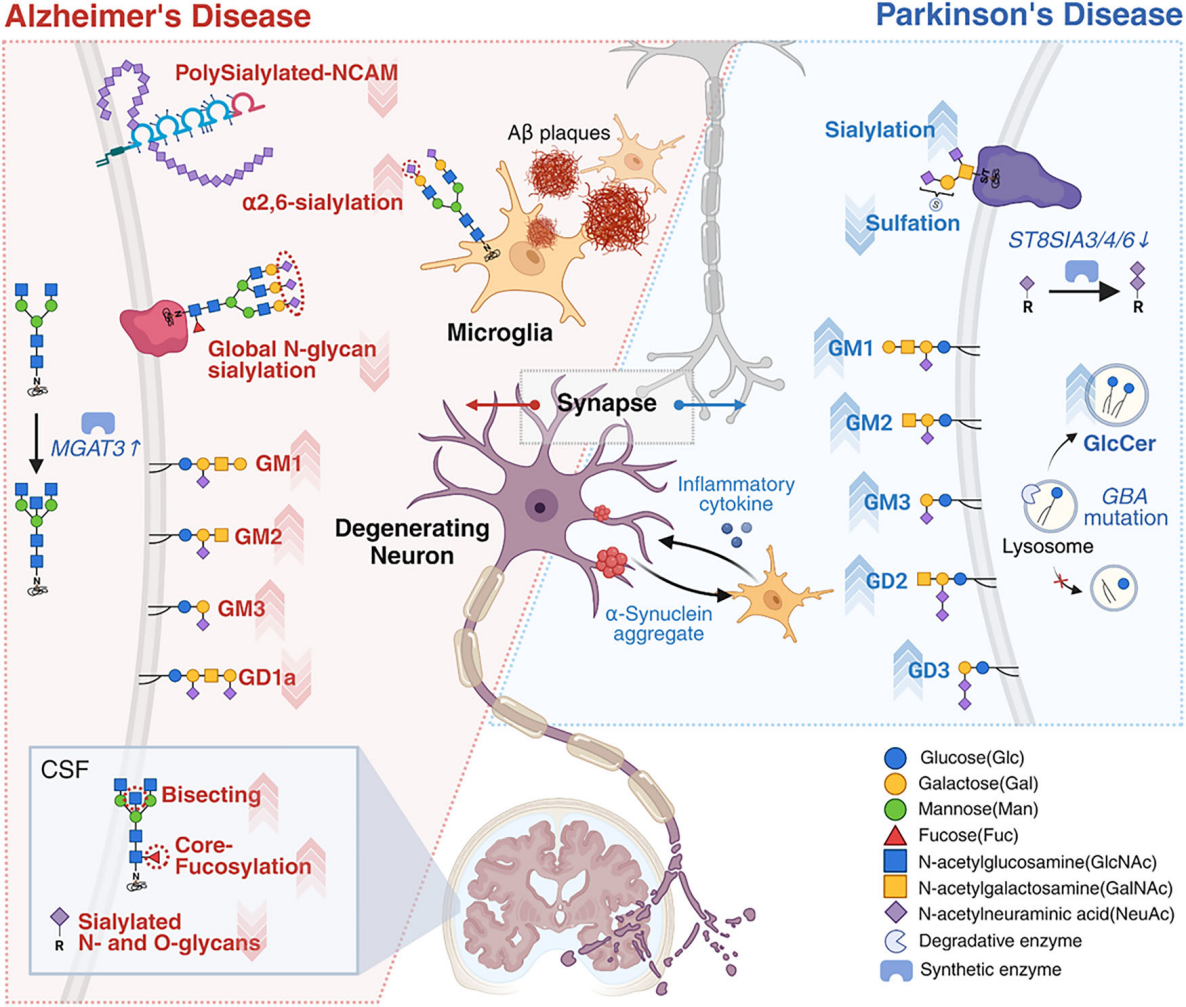
Alterations in brain glycosylation in Alzheimer’s and Parkinson’s diseases. In Alzheimer’s disease (AD) brains (red panel on the left), microglial N-glycan sialylation is increased, whereas overall glycoprotein sialylation and PSA-NCAM levels are reduced. A shift in ganglioside composition from GM1 to GM3 is also observed. Increased levels of bisecting and core-fucosylated N-glycans in CSF have been proposed as potential biomarkers for AD. In Parkinson’s disease (PD) brains (blue panel on the right), O-glycan sialylation is elevated, while sulfation is decreased. Ganglioside dysregulation is also evident, including lysosomal accumulation of GlcCer associated with GBA mutations, as well as overall increases in ganglioside levels. Created in BioRender. Park, J. (2025) https://BioRender.com/e5s15ha.

Neurological disorders encompass a wide range of diseases affecting the central and peripheral nervous systems, including neurodegenerative diseases such as AD and PD, as well as conditions such as epilepsy and stroke^67^. Among these, the association between AD and glycosylation has been one of the most extensively studied^56,58^ (Fig. 2 and Tables 1 and 2). AD is characterized by histopathological features closely linked to amyloid-beta (Aβ) production and aggregation^59,60,69^. In autopsied human brains, increased N-glycan sialylation has been observed in microglia, particularly around Aβ plaques^70^. Approximately 65% of microglia in plaque regions exhibit α-2,6-linked sialylation on their N-glycans, and elevated sialylation is also detected in nonplaque regions with high AD pathology^70^. Interestingly, high-throughput glycoproteomics analyses reveal an overall reduction in sialylation, especially in highly branched and elongated N-glycans^71^ (Fig. 2). Nonetheless, selective hypersialylation has been identified at specific sites, notably in clusterin (CLU), suggesting localized compensatory regulation. Polysialylated NCAM (PSA-NCAM), a key glycoprotein in neural plasticity, is generally reduced in patients with AD, particularly in the entorhinal cortex and hippocampus^62^ (Fig. 2). However, increased PSA-NCAM expression has also been reported in hippocampal subregions such as the dentate gyrus and CA1 in moderate-to-severe AD cases^72^, indicating region- and stage-specific dynamics during disease progression. Similar to observations in AD, O-glycan sialylation is also altered in the PD striatum^73^. This involves a marked increase in sialylation accompanied by a reduction in sulfation, suggesting that aberrant terminal glycosylation may disrupt ligand recognition by glycan-binding proteins such as sialic acid-binding immunoglobulin-type lectin 3 (Siglec-3) and Galectin-3 (a β-galactoside-binding lectin), as well as complement components such as C1q and Factor H. Such glycosylation defects may exacerbate microglial activation and increase synaptic vulnerability within the nigrostriatal pathway^73^ (Fig. 2).

Glycan profiling of cerebrospinal fluid (CSF) from patients with AD reveals elevated levels of bisecting and core-fucosylated N-glycans with reduced sialylation, probably associated with *N*-acetylglucosaminyltransferase III (MGAT3) overexpression^74^ (Fig. 2 and Table 1). O-glycans similarly show reduced sialylation. In PD, altered glycosylation has also been reported across multiple biological fluids. For example, urine N-glycan analysis shows reduced levels of biantennary galactosylated and sialylated structures^75^. By contrast, serum N-glycans display increased core fucosylation, bisecting GlcNAc and α2,6-linked sialylation^61^. These changes were detected not only at the protein level but also at specific glycosylation sites on 36 glycoproteins, including ceruloplasmin, haptoglobin, complement factor H and CLU, which are associated with neuroinflammatory and oxidative stress pathways.

In addition to protein-bound glycosylation, changes in brain ganglioside compositions, most notably GM1, GM2, GD1a and GD1b, have been implicated in both AD and PD, especially in connection with Aβ deposition and α-synuclein aggregation^76,77^ (Fig. 2 and Table 2). GM1 is known to bind Aβ peptide and facilitate its initial deposition, promoting plaque formation^76^. In human AD brain tissue, synaptic membranes from the precuneus show enhanced Aβ aggregation associated with ganglioside nanoclusters enriched in GM1, GD1 and GT1^76^. Region-specific ganglioside shifts have also been observed. GM1 d20:1 levels, as well as the GM1 d20:1/d18:1 ratio, are reduced in the dentate gyrus and entorhinal cortex^78^. Similarly, GM2 d20:1 and its d20:1/d18:1 ratio are decreased, whereas, GM2 and GM3 (d18:1) are enriched in Aβ plaque regions^79^. GM3, in particular, shows pronounced spatial co-localization with amyloid deposits, suggesting a pathological shift toward simpler gangliosides within plaque microenvironments. Recent advances in LC–MS-based glycolipidomics have enabled sensitive and comprehensive profiling of 184 ganglioside species including polysialylated forms, while minimizing in-source fragmentation^80^. When applied to the inferior frontal gyrus in AD brain, this approach uncovered elevated levels of simple gangliosides (GM2, GM3 and GQ3) and reduced levels of complex forms (GD1a, GT1b) (Fig. 2). Notably, GM3 (d20:1/18:0, d18:1/24:0, d18:0/18:0) and GQ3 (d18:1/18:0) showed high diagnostic potential as AD biomarkers.

Ganglioside dysregulation is also implicated in PD, particularly in patients with mutations in the lysosomal gene *GBA*, which accounts for up to 25% of PD cases^81^. These patients show elevated levels of GM1, GM2, GM3, GD2 and GD3 across multiple brain regions, including the middle temporal gyrus (MTG), cingulate gyrus (CG) and striatum^81^. Although glucosylceramide (GlcCer), the direct substrate of GBA, is only modestly increased in the MTG, ganglioside elevations are widespread. In addition, mutations in ATP10B, a lysosomal lipid flippase, impair GlcCer export and contribute to ganglioside accumulation, further implicating lysosomal dysfunction in PD pathogenesis^82^. Both GBA1 and ATP10B are now recognized as key regulators of GlcCer and ganglioside homeostasis.

Beyond AD and PD, ganglioside dysregulation has been implicated in other neurological conditions such as epilepsy and stroke (Table 2). In temporal lobe epilepsy, hippocampal profiling reveals increased levels of polysialylated gangliosides including GQ1b, GT1–3 and GQ2^83^. In particular, GQ1b is the dominant species and GQ2 is exclusively detected in temporal lobe epilepsy brain tissue. In addition, the elevated level of fucosylated and O-acetylated gangliosides suggest a distinct metabolic shift in seizure-related pathology. In the rat model of ischemic stroke, such as middle cerebral artery occlusion, GM1 ganglioside exhibits neuroprotective effects^84^. GM1 administration substantially reduces infarct volume and improves neurological function, particularly sensorimotor recovery. These therapeutic effects were associated with autophagy modulation, as indicated by decreased LC3-II and Beclin-1 and increased P62 expression.

### Psychiatric disorders

Psychiatric disorders, including MDD, PTSD and schizophrenia are characterized by diverse molecular and neurochemical alterations in the brain^85–87^. Emerging molecular studies on stress-related conditions including MDD and PTSD suggest that aberrant glycosylation contributes to their pathophysiology by disrupting neuronal signaling, synaptic plasticity and neuroimmune regulation^57,64,65^. In depression-like mouse models subjected to acute stress or chronic mild stress, hippocampal N-glycosylation is commonly altered, with both showing reduced bisecting glycans. However, chronic mild stress preferentially decreases multiantennary glycans, while acute stress primarily reduces high-mannose type glycans, indicating that distinct stress paradigms elicit divergent glycosylation responses in the hippocampus. In addition, enrichment analysis of glycoprotein-related genes, such as *Grin1*, *Grin2a*, *Thy1* and *Rcn1*, suggests that glycosylation changes may affect synaptic signaling and hippocampal function in depression^57^. Consistently, increased O-GlcNAcylation in astrocytes of the medial prefrontal cortex (mPFC) has been shown to modulate glutamatergic transmission, and thus affect stress susceptibility and depressive-like behaviors^65^. Similarly, trauma-induced N-glycosylation changes have been reported in the prefrontal cortex (PFC), where core-fucosylated biantennary glycan was increased in stress-susceptible animals, accompanied by a shift toward simpler and less sialylated glycans. These findings support a potential link between glycosylation patterns and vulnerability to stress^64^. Collectively, these results exhibit protein-bound glycosylation in the brain as a critical modulator of psychiatric disorders.

In parallel, brain gangliosides, a class of sialylated glycolipids enriched in neuronal membranes, have also been implicated in depression and PTSD^54,88,89^. These molecules are key regulators of synaptic stability, neuroinflammation and signal transduction^89,90^. Among them, ganglioside GD3 is essential for maintaining quiescent neural stem cells and supporting adult neurogenesis in neurogenic regions such as the subventricular zone and dentate gyrus^89^. Mice lacking GD3 exhibit reduced neurogenesis, memory and olfactory impairments, and depressive-like behaviors^54^.

Although most of these findings are derived from animal models exhibiting stress-related behaviors, they underscore the need for translational studies in human populations^91–95^. Recent efforts have expanded glycosylation research to human fluids such as blood, CSF and extracellular vesicles (EVs) to identify novel biomarkers and therapeutic targets for psychiatric disorders^91,92,94–96^. In schizophrenia, distinct N-glycosylation profiles have been observed including elevated polylactosaminylated glycans and SLe^x^-containing glycans in serum from male patients, and reduced bisecting and sialylated glycans in CSF, suggesting potential sex-specific glycosylation^96^. Similarly, in MDD, altered glycosylation patterns in plasma and EVs have been reported such as reduced levels of α2,6-sialylated glycans and decreased wheat germ agglutinin (WGA)-binding GlcNAc and sialic acid signals^92^. Notably, the largest genome-wide association study meta-analysis of MDD (~688,000 cases) identified several glycosylation-related genes including *GALNT*s among genetic loci associated with antidepressant response, further supporting the role of glycosylation in psychiatric phenotypes^93^. Together, these results emphasize the therapeutic relevance of glycosylation in psychiatric disorders and highlight its potential for guiding biomarker discovery and treatment stratification.

### Neurodevelopmental disorders

**Fig. 3:**
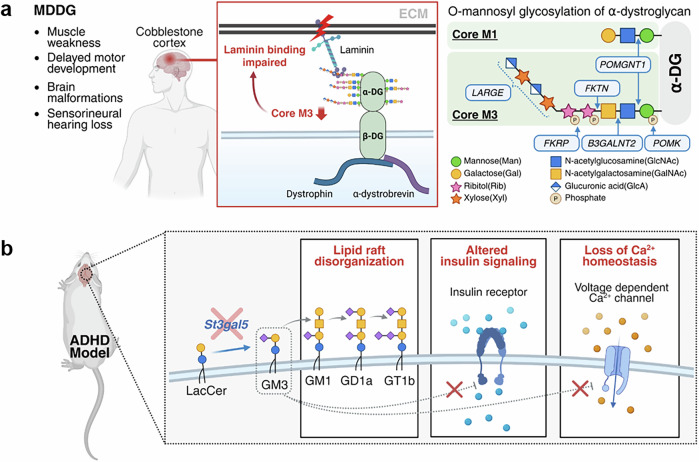
Alterations in brain glycosylation in neurodevelopmental disorders. **a** In MDDG, defective O-mannosylation of α-DG impairs laminin binding and reduces Core M3 and matriglycan (GlcA–Xyl) synthesis, disrupting cell–ECM interactions and causing muscle symptoms. **b** *St3gal5*-deficient mice with ADHD-like behaviors show loss of GM3-derived gangliosides, disrupted lipid rafts and altered insulin and calcium signaling. Created in BioRender. Park, J. (2025) https://BioRender.com/iwxja8y.

Neurodevelopmental disorders including autism spectrum disorder, muscular dystrophy-dystroglycanopathy (MDDG) and attention deficit hyperactivity disorder (ADHD) have increasingly been studied in the context of glycosylation, although research in this area remains limited^31,97–100^. Nevertheless, these early findings underscore the potential role of glycosylation in the pathophysiology of these conditions. MDDG is characterized by defective O-mannosyl glycosylation of α-DG, resulting in impaired laminin binding and subsequent disruption of neuron–glia–ECM interactions^97,100^. Among the causative glycosyltransferases, mutations in the B3GALNT2 gene impaired the glycosylation of α-DG by affecting Core M3 formation. This disruption prevented the synthesis of the functional matriglycan (GlcA–Xyl repeat), which mediates laminin binding, and ultimately led to congenital muscular dystrophy with variable brain and ocular involvement^100^ (Fig. 3a). In ADHD, changes in serum glycosylation have also been reported, including increased antennary fucosylation, reduced bisecting GlcNAc on di-/triantennary N-glycans and decreased α2,3-sialylation^98^. ADHD and anxiety disorders share overlapping symptoms, such as restlessness, difficulty concentrating and irritability, despite potentially distinct underlying molecular mechanisms^101–104^. While clinical diagnosis based on behavioral and symptomatic evaluations is the current standard, such assessments can be ambiguous in differentiating between overlapping conditions. Therefore, there is a growing need for molecular biomarkers to enable more precise classification and targeted treatment. These glycosylation alterations suggest the potential utility of glycosylation-based biomarkers for ADHD and further highlight the involvement of glycosylation in neurodevelopmental dysregulation.

Disruptions in ganglioside metabolism have likewise been implicated in neurodevelopmental disorders. In mouse models showing ADHD-like behaviors, deficiency of *St3gal5*, which impairs the synthesis of GM3 ganglioside, a precursor for complex gangliosides such as GM1, GD1a and GT1b, led to hyperactivity and anxiety-like behaviors^105^ (Fig. 3b). These phenotypes were accompanied by impaired insulin receptor signaling and electroencephalography abnormalities, highlighting the role of GM3-related gangliosides in both behavioral regulation and metabolic signaling. In autism spectrum disorder, reduced plasma levels of sialic acid suggest potential defects in ganglioside biosynthesis, while elevated levels of anti-GM1 antibody indicate an immune-mediated disruption of neuronal ganglioside function^99^. These findings collectively highlight the critical role of ganglioside metabolism in neurodevelopmental processes and its potential contribution to their pathophysiology.

## From mechanisms to medicine: future perspectives in brain glycosylation

**Fig. 4:**
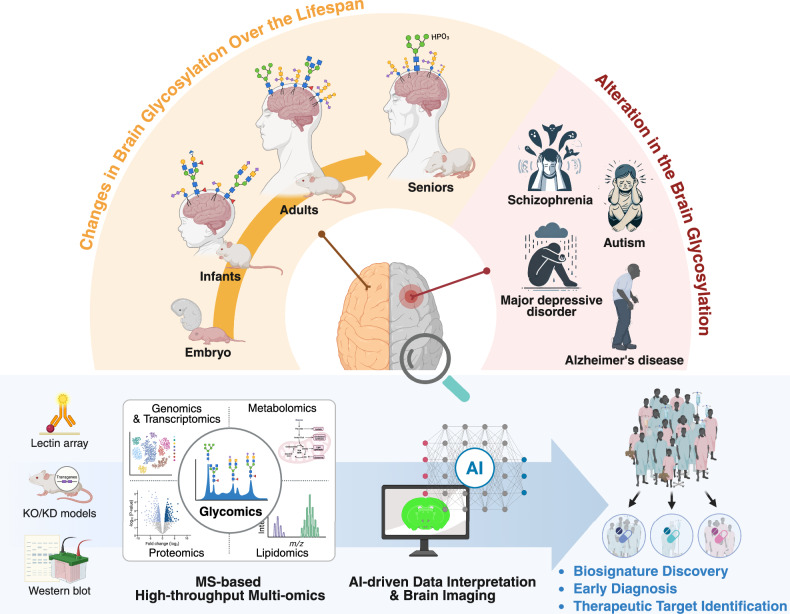
Lifespan and disease-associated changes in brain glycosylation and omics-based analysis. Brain glycosylation dynamically changes across development and aging. Temporal shifts in glycan profiles occur naturally over the lifespan and can also be driven by brain disorders. Analytical strategies are evolving from conventional tools to multi-omics and AI-based approaches, enabling deeper insights into brain pathology. KD, knockdown; KO, knockout. Created in BioRender. Park, J. (2025) https://BioRender.com/h8mnghu.

Glycosylation contributes substantially to a broad range of developmental and physiological processes from embryogenesis and organogenesis to aging^12,35,106^ (Fig. 4). It is essential for cell–cell recognition, adhesion and signal transduction, thereby maintaining the structural and functional integrity of tissues and organs^17^. Dysregulated or aberrant glycosylation, whether driven by genetic mutations or altered physiological conditions, is closely associated with the onset and progression of various diseases, including congenital disorders, cancers and age-related neurodegenerative conditions^17,107^.

In the brain, mounting evidence from molecular neuroscience has identified glycosylation as a key regulatory mechanism that governs numerous dynamic cellular interactions within the neural microenvironment^17^. These include neuron–neuron communication, neurotransmitter transport, neuron–astrocyte coordination and glia–endothelial interactions that regulate the blood–brain barrier. Such glycosylation-dependent mechanisms are central to decoding the complexity of brain circuits and understanding both the intrinsic physiology and pathological changes in the central nervous system. Increasingly, glycosylation is being recognized not as a static posttranslational modification, but as a dynamic and spatially regulated process critical for interregional functional connectivity in the brain^15,16^. Indeed, recent studies have further demonstrated that brain glycosylation characteristics are highly dependent on species, age and anatomical subregions^12,13,22,24,56^. Furthermore, interest is growing in the glycosylation patterns of distinct brain cell types, such as glutamatergic and GABAergic neurons, astrocytes and oligodendrocytes. Together, these insights reinforce the need for in-depth investigations of region- and cell-type-specific glycosylation across different mammalian brains, as well as studies aimed at dissecting their distinct functional roles in the neural physiology and disease.

Historically, brain glycosylation studies have relied on targeted approaches including gene knockout and knockdown models, western blotting and lectin-based array techniques^70,72,89,95^. While these techniques have provided important insights into specific glycan moieties and biosynthetic enzymes, they fall short in capturing the full complexity and diversity of the brain glycome (Fig. 4). Recent methodological breakthroughs, particularly in glycan-specific enrichment strategies and high-resolution MS, have revolutionized the field. Advanced MS/MS techniques such as stepped collision-induced dissociation and electron-transfer/higher-energy collision dissociation now enable comprehensive structural characterization of N- and O-glycans, as well as glycolipids^13,26,71^. Integration with LC and capillary electrophoresis enhances separation power, while matrix-assisted laser desorption/ionization (MALDI)-based MS imaging (MSI) allows spatial mapping of glycosylation patterns across brain regions, bridging molecular information with histological context^13,79^.

Looking forward, the development of large-scale, glycomics-centered multi-omics strategies will be crucial. Integrating glycomics with genomics, transcriptomics, proteomics and metabolomics will allow for a more comprehensive understanding of brain function and disease. These platforms will enable genome-wide association study-inspired, population-level studies to investigate the biological importance of glycosylation heterogeneity and its associations with genetic background, physiological traits and disease susceptibility. In parallel, efforts to construct brain region-specific and cell-type-specific glycome atlases and reliable databases will provide critical resources for both basic and translational research. Moreover, glycomics-centered multi-omics strategies including glycoproteomics, glycolipidomics and glycoengineering are poised to uncover previously unrecognized pathological mechanisms in complex brain disorders (Fig. 4).

At the same time, the enormous structural diversity and biosynthetic complexity of glycans remain a major bottleneck for data interpretation. To overcome these challenges, AI-driven bioinformatics and machine learning approaches are becoming indispensable^87,88^. These technologies facilitate the analysis of high-dimensional glycome datasets and enable the integration of glycan profiles with transcriptomic, proteomic and clinical datasets, thereby unlocking new layers of biological insight^108–110^ (Fig. 4).

Eventually, the construction of a brain glycome atlas and publicly accessible database will play a critical role, much like the Human Protein Atlas, in supporting and complementing global brain research initiatives such as the HUMAN Brain Project, the BRAIN Initiative Cell Census Network, the Human Proteome Project and emerging efforts toward a Brain Proteome Project. These progressively advancing projects would facilitate the integration of glycomics with multi-omics data and advance our systematic understanding of glycosylation in both normal brain function and neurological diseases.

In conclusion, brain glycosylation represents a rapidly expanding frontier in molecular neuroscience with transformative implications for decoding brain function, understanding disease mechanisms and advancing therapeutic development. Its relevance spans a wide spectrum of disorders from neurodegenerative diseases such as AD and PD to psychiatric disorders including MDD, PTSD and schizophrenia, which impose a substantial global health burden. Unlocking the diverse and dynamic roles of glycosylation in the brain is therefore essential for the development of next-generation diagnostics and precision therapeutics. Moving forward, continued advancements in analytical technologies, large-scale glycomics datasets and convergence with systems-level computational approaches will be pivotal in bridging the gap between basic glycoscience and clinical neuroscience.
